# Flexural Wave Propagation in Mass Chain-Filled Carbon Nanotubes

**DOI:** 10.3390/ma12182986

**Published:** 2019-09-15

**Authors:** Rumeng Liu, Junhua Zhao, Lifeng Wang

**Affiliations:** 1 Jiangsu Key Laboratory of Advanced Food Manufacturing Equipment and Technology, Jiangnan University, Wuxi 214122, China; rmliu@jiangnan.edu.cn; 2 State Key Laboratory of Mechanics and Control of Mechanical Structures, Nanjing University of Aeronautics and Astronautics, Nanjing 210016, China; walfe@nuaa.edu.cn

**Keywords:** carbon nanotube, wave propagation, carbon-atom chain, van der Waals interaction

## Abstract

The propagation characteristics of terahertz (THz) flexural waves in mass chain-filled single-walled carbon nanotubes (MCSCs) are studied using a continuum mechanics approach and molecular dynamics (MD) simulations, where each single-walled carbon nanotube (SWCNT) is modeled as a nonlocal Timoshenko beam based on the nonlocal strain gradient theory. The effect of the surrounding elastic medium and the van der Waals (vdW) interactions between the mass chain and the SWCNT on the wave propagation is quantitatively considered in governing equations, respectively. The analytical expressions of two flexural wave branches and the bandgap between the two branches are derived. When combining our MD simulations of the carbon-atom chain-filled SWCNT, the wave within the bandgap disperses rapidly, and the mass chain has a significant influence on the phase velocity of the flexural wave. The present theoretical solution has a high accuracy in a wide frequency range up to the THz region. In particular, the surrounding elastic medium of the MCSCs remarkably affects the phase velocity for low frequencies, but not for high frequencies. The present study indicates that the wave propagation of a SWCNT could be modulated by changing the filled mass chain and the surrounding elastic medium.

## 1. Introduction

Carbon nanotubes (CNTs) hold attractive potentials for semiconductor transducers and resonator devices because of their excellent electronic conductivity and mechanical strength [1,2,3,4,5,6,7,8,9]. CNT resonators with a reachable ultrahigh frequency range up to the terahertz (THz) order provide a platform for exploring a broad range of physical phenomena [10,11,12,13,14,15,16]. Thus, in the past few years, the dynamic behaviors of individual CNTs have been systematically investigated, using experimental methods, first-principles calculations, molecular dynamics (MD) simulations, and continuum modeling. Tsioutsios et al. reported a sensitive method to resolve the mechanical fluctuations of CNT resonators in real-time using the electron microscope [17]. Taking the microstructure effect of CNTs into consideration, Wang and Hu established a nonlocal elastic shell model [18] and a nonlocal beam model [19] to investigate both longitudinal and flexural wave propagation in single-walled carbon nanotubes (SWCNTs), respectively. By considering the interactions between two neighboring SWCNTs, sound wave propagation in individual multi-walled CNTs [20] and double-walled CNTs [21] were studied by the beam model. Li et al. analyzed the effect of a surrounding elastic medium on flexural wave propagation in CNTs via the nonlocal Timoshenko beam model [22].

On the other hand, the CNT is also one of the most promising nanomaterials for assembling nanocomposite structures, such as fluid-filled CNTs [23,24], metal nanowire-filled SWCNTs [25,26], carbon-atom chain-filled SWCNTs (CCSCs) [27,28,29], and C_60_ chain-filled SWCNTs [30]. It is well-known that the van der Waals (vdW) interactions between the filled mass chain and the CNT play an important role in the dynamics behavior of the CNT composite structures. Yang et al. established a dynamic Timoshenko beam model to study the dynamic behavior of fluid-filled CNTs [31]. The bucking behavior and vibration of CCSCs were studied using continuum modeling and MD simulations [32,33,34,35,36]. Recently, Lu et al. have found the metamaterial-like vibrations in the CCSCs and C_60_ chain-filled SWCNTs using the classical Euler beam model [37]. The similar dynamic metamaterial-like vibration behaviors of double-walled CNTs have been reported by Yoon and Ru [38]. Despite the fact that the dynamic properties of CNTs and CNT composite structures have been extensively studied in the available literature, the flexural wave propagation characteristic in mass chain-filled CNTs is still not clear. In particular, the effect mechanism of the CNT microstructures and the surrounding elastic medium on their wave propagation remains unknown, and should be further revealed.

The main objective of this work is to present a detailed study on the flexural wave propagation in mass chain-filled single-walled carbon nanotubes (MCSCs). In Section 2, a nonlocal Timoshenko beam model is established to describe the dynamic behavior of MCSCs, where the effect of the surrounding elastic medium and the vdW interactions between the CNT and the filled mass chain on the wave frequency is included by considering the effect of the CNT microstructures. The MD simulations used to check the results of the continuum modeling are outlined in Section 3. In Section 4, the obtained MD results are further discussed by comparison with those from the nonlocal Timoshenko beam model. Finally, some concluding remarks are made in Section 5.

## 2. Nonlocal Timoshenko Beam Model of MCSCs

**Figure 1:**
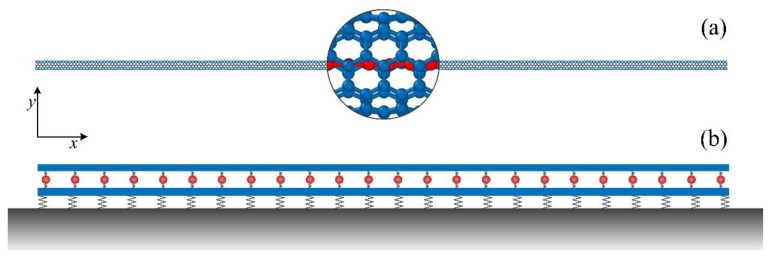
The mass chain-filled single-walled carbon nanotubes (SWCNTs) system. (**a**) Molecular structure model of a carbon-atom chain in a (5, 5) SWCNT; (**b**) continuum model of a mass chain-filled single-walled carbon nanotube (MCSC) system embedded in an elastic medium, the hollow cylindrical beam in blue is the equivalent model of a SWCNT.

This section starts at the coupled dynamics equations of a MCSC system with an infinite length along the *x* direction in the frame of Cartesian (*x*, *y*, *z*) coordinates. Taking the CCSC as an example, Figure 1a shows the molecular structure of a carbon-atom chain in a (5, 5) SWCNT. Considering the effect of the CNT microstructures, the SWCNT is modeled as a nonlocal Timoshenko beam, while the mass chain is modeled as a string with no bending rigidity. As shown in Figure 1b, the vdW interactions between the mass chain and the SWCNT are taken as linear springs with the stiffness coefficient (per unit length) of $c_{12}$. Here, the effect of the surrounding elastic medium on the MCSC is also considered, using linear springs with the stiffness coefficient of $c_{13}$. Therefore, the coupled governing equations for the MCSC are given by:

(1) $$\rho A\frac{\partial^{2}w_{1}}{\partial t^{2}}+\beta GA\left[\left(\frac{\partial \varphi_{1}}{\partial x}-\frac{\partial^{2}w_{1}}{\partial x^{2}}\right)+r^{2}\left(\frac{\partial^{3}\varphi_{1}}{\partial x^{3}}-\frac{\partial^{4}w_{1}}{\partial x^{4}}\right)\right]=c_{12}\left(w_{2}-w_{1}\right)-c_{13}w_{1},\;\rho I\frac{\partial^{2}\varphi_{1}}{\partial t^{2}}+\beta GA\left[\left(\varphi_{1}-\frac{\partial w_{1}}{\partial x}\right)+r^{2}\left(\frac{\partial^{2}\varphi_{1}}{\partial x^{2}}-\frac{\partial^{3}w_{1}}{\partial x^{3}}\right)\right]-EI\left(\frac{\partial^{2}\varphi_{1}}{\partial x^{2}}+r^{2}\frac{\partial^{4}\varphi_{1}}{\partial x^{4}}\right)=0,\;\rho_{c}\frac{\partial^{2}w_{2}}{\partial t^{2}}=c_{12}\left(w_{1}-w_{2}\right),$$

where $w_{1}\left(x,t\right)$ and $w_{2}\left(x,t\right)$ are the displacements of section *x* of the SWCNT and the mass chain along the *y* direction at moment *t*, respectively; $\varphi_{1}$ is the slope of the deflection curve of the SWCNT; and I and β are the moment of inertia and the form factor, depending on the shape of the cross-section of the SWCNT, respectively. E, ρ, A, and G are Young’s modulus, mass density, the cross-section area, and the shear modulus of the SWCNT, respectively; $\rho_{c}$ is the linear density of the mass chain; r is a small-scale parameter describing the effect of the CNT microstructure on elastic behavior; and it yields [19]:

(2) $$r=\frac{L_{c}}{\sqrt{12}},$$

where $L_{c}=0.123\mathrm{nm}$ is the axial distance between two rings of carbon atoms for the armchair SWCNT.

For an infinitely long MSCS system, the dynamic deflection and slope are given by [19]:

(3) $$w_{1}=\hat{w}e^{jk\left(x-ct\right)},\;\varphi_{1}=\hat{\varphi }e^{jk\left(x-ct\right)},$$

where $j\equiv \sqrt{-1}$. The relationship between the phase velocity c, the wave number k, and the frequency ω of the flexural wave follows:

(4) $$k=\frac{\omega }{c}.$$

Substituting Equation (3) into Equation (1), and eliminating $w_{2}$, one can obtain the following equation:

(5) $$\left(\begin{array}{cc} \Delta_{1} & \Delta_{1}\\ \Delta_{1} & \Delta_{1} \end{array}\right)\left(\begin{array}{c} \hat{w}\\ \hat{\varphi } \end{array}\right)=0,$$

where:

(6) $$\left\{ \begin{array}{l} \Delta_{1}=\bar{A}\Omega^{2}+\bar{B}\Omega +c_{12}k\bar{D}+c_{12}c_{13},\\ \Delta_{2}=c_{12}j\bar{D}-\rho_{c}j\Omega \bar{D},\\ \Delta_{3}=-j\bar{D},\\ \Delta_{4}=-\rho I\Omega +\bar{C}, \end{array}\right.$$

where:

(7) $$\left\{ \begin{array}{l} \bar{A}=\rho_{c}\rho A,\\ \bar{B}=\rho_{c}\beta AGk^{2}\left(r^{2}k^{2}-1\right)-\left(c_{13}\rho_{c}+c_{12}\rho_{c}+c_{12}\rho A\right),\\ \bar{C}=\left(\beta AG+EIk^{2}\right)\left(1-r^{2}k^{2}\right),\\ \bar{D}=\beta AGk\left(1-r^{2}k^{2}\right),\\ \Omega =\omega^{2}. \end{array}\right.$$

Thus, the following equation can be obtained from the fact that at least one nonzero solution $\left(\hat{w},\hat{\varphi }\right)$ of Equation (5) exists:

(8) $$\Delta_{1}\Delta_{4}-\Delta_{2}\Delta_{3}=0.$$

It is noticed that Equation (8) is a six-order equation of ω, and it gives three branches of wave dispersion relation, with the lower two branches representing the dispersion relation of the flexural wave. Letting $c_{13}=0$, Equation (8) leads to the dynamic equation of an independent MCSC system without the surrounding elastic medium. If $\rho_{c}=0$, Equation (1) leads to the wave dispersion relation of the SWCNT:

(9) $${\bar{A}}_{1}c^{4}-{\bar{B}}_{1}c^{2}+{\bar{C}}_{1}=0,$$

where:

(10) $$\left\{ \begin{array}{l} {\bar{A}}_{1}=\frac{\rho_{}^{2}Ik^{2}}{\beta G},\\ {\bar{B}}_{1}=\left[\rho A+\left(\frac{E}{\beta G}+1\right)\rho Ik^{2}\right]\left(1-r^{2}k^{2}\right)+\left[\frac{c_{13}}{\beta GA}\right]\rho I,\\ {\bar{C}}_{1}=EIk^{2}{\left(1-r^{2}k^{2}\right)}^{2}+\left(\frac{EIk^{2}}{\beta GA}+1\right)\frac{c_{13}}{k^{2}}\left(1-r^{2}k^{2}\right). \end{array}\right.$$

## 3. MD Simulations for Flexural Wave Propagation in CCSC

**Figure 2:**
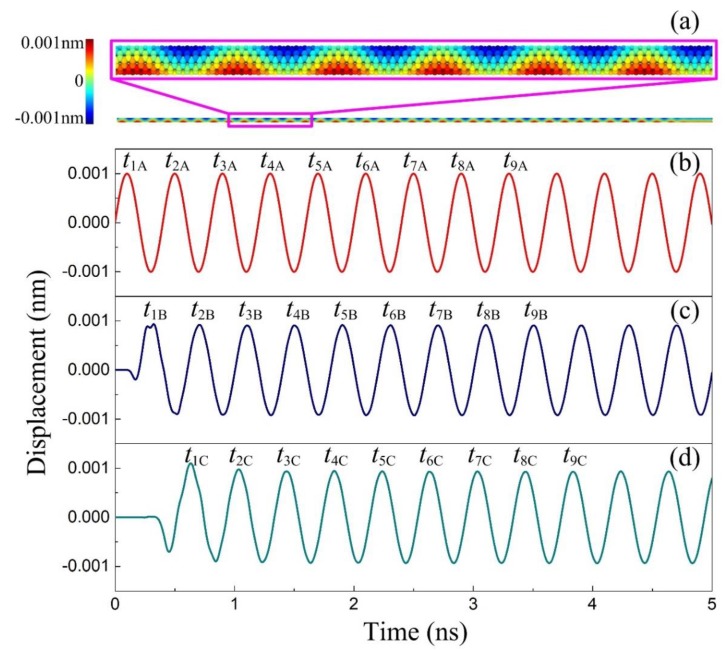
Molecular dynamics (MD) simulations of flexural wave propagation in a carbon atom chain-filled SWCNT (CCSC). (**a**) A snapshot of the displacement nephogram in the *y* direction of the (5, 5) SWCNT; (**b**) the input wave with frequency *f* = 2.5 THz at the edge layer of the SWCNT, where subscripts *i* in *t_iA_* represent the number of the wave peak; (**c**) the wave at section B, which is 2.25 nm ahead of section A; (**d**) the wave at section C, which is 4.69 nm ahead of section A.

The present MD simulations are performed to investigate the dispersion relations of the CCSC system. The Brenner’s second generation reactive empirical bond order (REBO) potential [39], which has been widely used to study the mechanical behaviors of carbon materials, is applied to describe the interatomic interactions, while the long-range vdW interactions between the SWCNT and the carbon-atom chain are calculated by the LJ 12-6 potential, which is given by:

(11) $$E_{\mathrm{vdW}}=4\varepsilon \left[{\left(\frac{\sigma }{r}\right)}^{12}-{\left(\frac{\sigma }{r}\right)}^{6}\right],$$

with the well-depth energy $\varepsilon =2.84\times 10^{-3}\mathrm{eV}$, and the equilibrium distance of σ=0.34nm for the carbon atoms. For the carbon-atom chain-(5, 5) SWCNT system, the length of the carbon-atom chain and SWCNT are both taken as 123 nm. In the MD simulations, an energy minimization of the structures is firstly performed by the steepest descent algorithm. After relaxation, the positions (along the *y* direction) of the first layer of atoms in the SWCNT are shifted following Asin(ωt) to achieve the harmonic deflection while the remaining atoms part, and the carbon-atom chain is allowed to evolve freely in the *NVE* ensemble. Then, the flexural wave will propagate in the SWCNT, as shown in Figure 2a. The harmonic deflection of the edge layer is shown in Figure 2b, while Figure 2c,d display the flexural vibrations of section B at $x_{1}=2.25\mathrm{nm}$ and section C at $x_{2}=4.69\mathrm{nm}$, respectively. To determine the phase velocity, the propagation duration Δt of the flexural wave from section B to section C can be obtained by:

(12) $$\Delta t\approx \frac{\left(t_{3C}-t_{3B}\right)+\left(t_{4C}-t_{4B}\right)+\cdots +\left(t_{nC}-t_{nB}\right)}{n-2},$$

where the first two periods are neglected. Then, the phase velocity can be determined by [19]:

(13) $$c=\frac{x_{2}-x_{1}}{\Delta t}.$$

## 4. Results and Discussion

First, to predict the flexural wave propagation in the CCSC, it is necessary to know the material and geometrical properties of the SWCNT and the carbon-atom chain. Here, the Young’s modulus E=0.80TPa, the Poisson’s ratio μ=0.254, the mass density $\rho =2237\mathrm{kg}/m^{3}$, and the diameter d=0.678nm for the armchair (5, 5) SWCNTs, when the thickness of the wall is chosen as 0.34 nm. The linear density of the carbon-atom chain is defined by $\rho_{c}=m_{c}/d_{c}$, where $m_{c}$ is the mass of one single carbon atom, and $d_{c}=0.1229\mathrm{nm}$ is the distance between two adjacent carbon atoms [37]. The vdW coefficient $c_{12}$ between the carbon-atom chain and the (5, 5) SWCNT is taken as 111 GPa [34].

**Figure 3:**
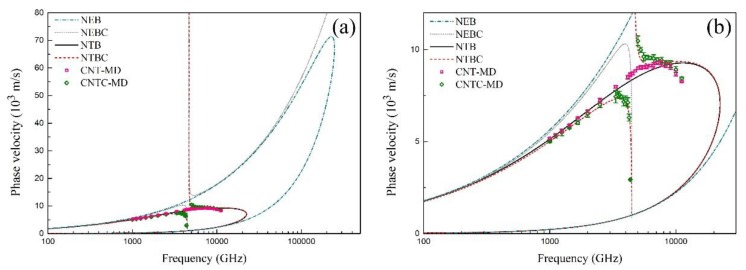
The dispersion relation of the SWCNT and the CCSC obtained by different beam models and MD simulations. (**a**) The dependence of the phase velocity of the sinusoidal flexural wave on the wave frequency; (**b**) the zoom of (**a**).

Figure 3a displays the dispersion relations between the phase velocity and the wave frequency f=ω/2π of the flexural wave in the SWCNT and the CCSC, which is obtained from the beam model and the MD simulations. Here, the symbol NEB represents the nonlocal Euler beam model; NEBC represents the nonlocal Euler beam with carbon-atom chain model; NTB represents the nonlocal Timoshenko beam model; NTBC represents the nonlocal Timoshenko beam with carbon-atom chain model; CNT-MD represents the MD results for SWCNT; and CNTC-MD represents the MD results for the CCSC, respectively.

**Figure 4:**
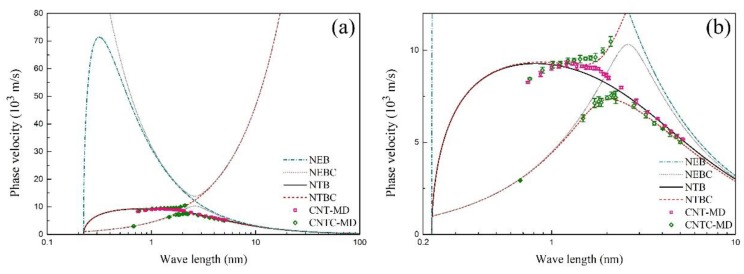
(**a**) The dependence of the phase velocity on the wave length of the SWCNT and the CCSC; (**b**) the zoom of (**a**).

**Figure 5:**
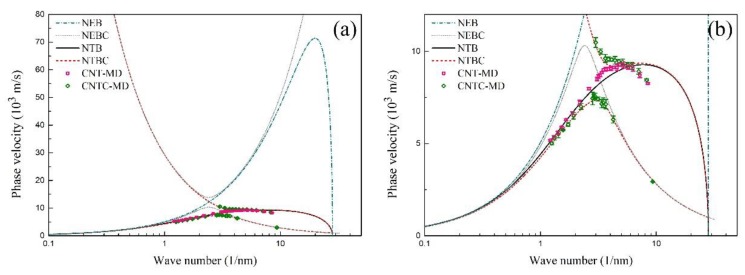
(**a**) The phase velocity versus the wave number of the SWCNT and the CCSC; (**b**) the zoom of (**a**).

**Figure 6:**
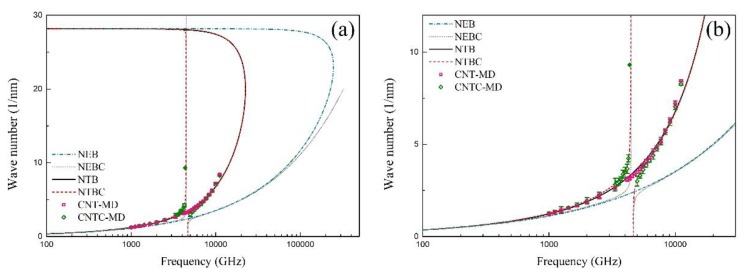
(**a**) The dependence of the wave number on the wave number of the SWCNT and the CCSC; (**b**) the zoom of (**a**).

It can be found from Figure 3a that the NEB model can predict the MD results well when the wave frequency is approximately smaller than 0.4 THz. The difference between the NEB model and the MD results becomes more obvious with the increasing wave frequency. However, the results from the NTB model and NTBC model coincide with the MD results well in a wide frequency range. Furthermore, Figure 3b shows the zoom of Figure 3a, where the phase velocity of the SWCNT and the CCSC are close to each other when the wave frequency is smaller than approximately 1 THz. In the range from 1 THz to 3.7 THz, both the phase velocities of the SWCNT and the CCSC increase with the increasing wave frequency, where the phase velocity of the SWCNT is bigger than that of the CCSC. In particular, the phase velocity of the CCSC suddenly decreases when the wave frequency is bigger than 3.7 THz, while no results about the phase velocity can be obtained from Equation (8), when the wave frequency is within a bandgap from 4.47 THz to 4.67 THz. Next, a higher branch of the CCSC occurs when the wave frequency is larger than 4.67 THz. The propagation of flexural waves in both the SWCNT and the CCSC is blocked by the effect of the CNT microstructure when the wave frequency exceeds 7 THz, where the wave number is very large and the wave length is very short, as shown in Figure 4, Figure 5 and Figure 6. By contrast with the results of the MD simulations, Figure 3 shows that the flexural wave dispersion in the SWCNT and the CCSC by the NTB model and the NTBC model has a high accuracy in a wide frequency range.

**Figure 7:**
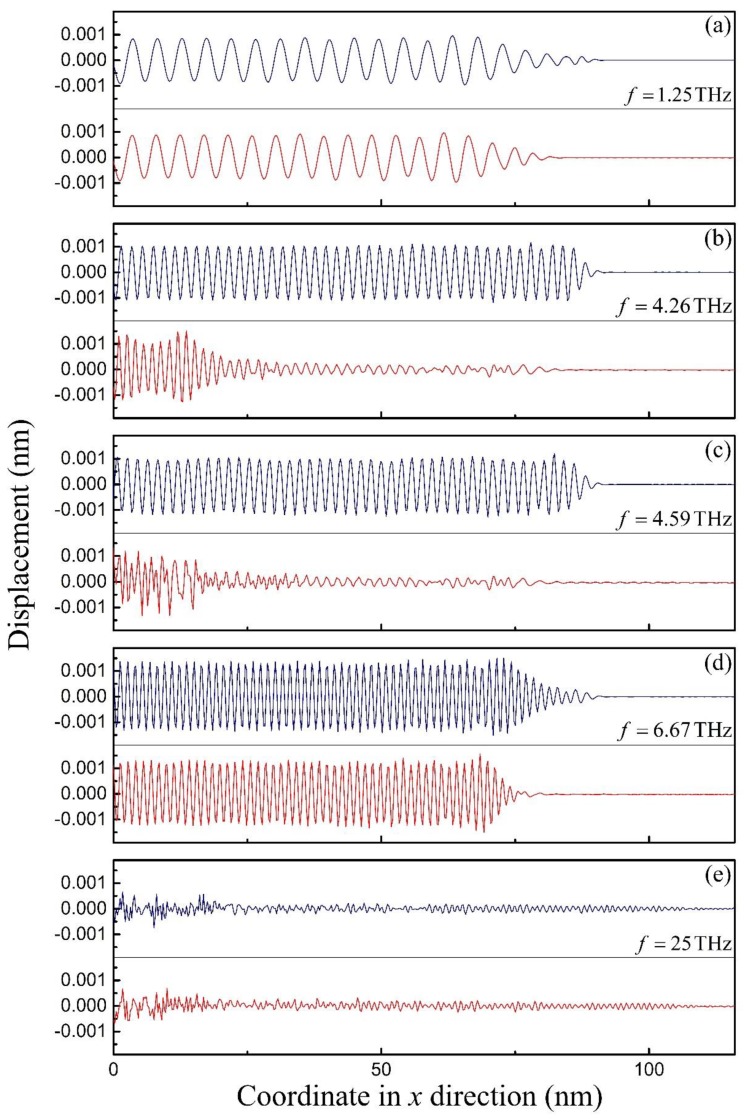
The propagation of waves of frequencies of (**a**) 1.25 THz, (**b**) 4.26 THz, (**c**) 4.59 THz, (**d**) 6.67 THz, and (**e**) 25 THz, respectively, in a (5, 5) SWCNT (solid line in blue) and a CCSC (solid line in red) at 80 ps, simulated by MD simulations.

Furthermore, by taking the moment of 80 ps in the MD simulations as an example, the flexural wave propagation in the SWCNT and the CCSC are displayed in Figure 7, which demonstrates the dispersion relations between the phase velocity and the wave frequency. At the wave frequency f=1.25THz, Figure 7a shows that the wave propagates steadily in both the SWCNT and the CCSC with similar wave modes, which means that the phase velocities in these two structures are close to each other. As the frequency increases to 4.26 THz, the difference of the wave modes between these two structures becomes remarkable, as shown in Figure 7b. Obviously, Figure 7c shows that the wave can hardly propagate in the CCSC at f=4.59THz, which is within the bandgap. When the wave frequency surmounts the bandgap, the wave can propagate in the CCSC again, and it shows similar wave modes to the SWCNT, as displayed in Figure 7d. As illustrated in Figure 7e, the wave modes of these two structures are both irregular, and the wave dispersed rapidly at the high frequency f=25THz, due to the effect of the SWCNT microstructure.

**Figure 8:**
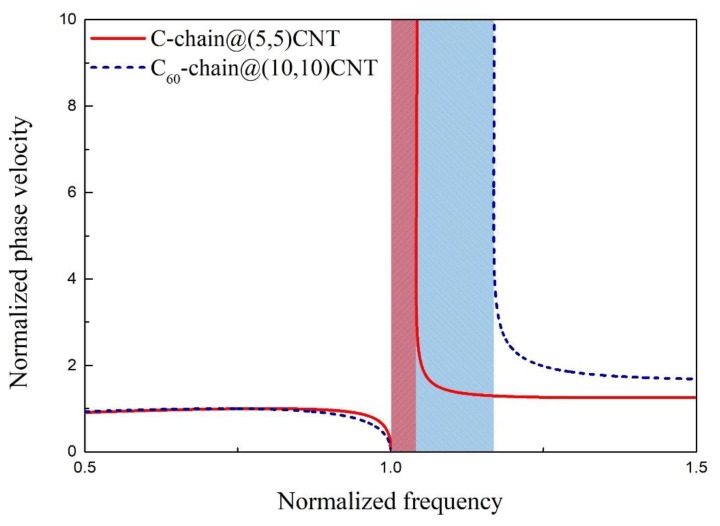
The normalized phase velocity versus the normalized wave frequency of the CCSC and the C60 chain-filled SWCNT. The rectangular shadow in red and blue represents the bandgap of the CCSC and the C60 chain-filled SWCNT, respectively.

From the present nonlocal Timoshenko beam model, the bandgap width of the MCSC is related with the linear density of the filled mass chain, and the vdW interaction between the mass chain and the SWCNT. Figure 8 shows the distribution of the normalized phase velocity with respect to the normalized wave frequency of the CCSC and a (10, 10) SWCNT filled with a C_60_ chain. Here, the distance between two adjacent C_60_ is 1 nm, the linear density of the C_60_ chain is $1.2\times 10^{-15}\mathrm{kg}/m$, and the vdW coefficient between the C_60_ chain and the SWCNT is 76 GPa [37]. It is clear that the bandgap width of the C_60_ chain-filled (10, 10) SWCNT is wider than that of the CCSC.

**Figure 9:**
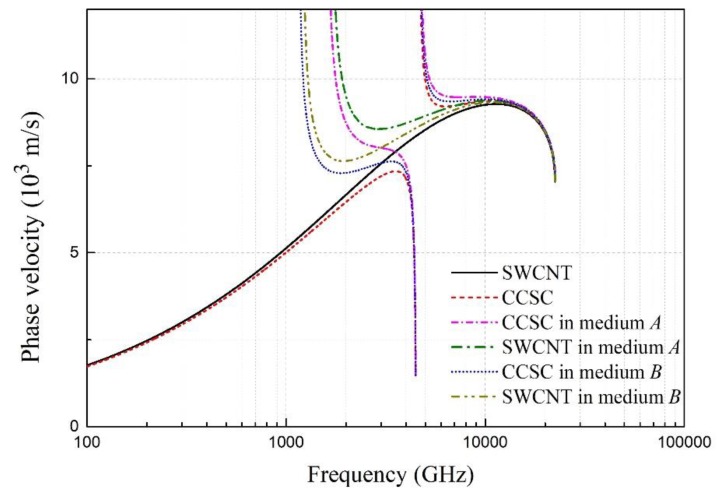
The phase velocity of the flexural wave versus the frequency of the SWCNT and the CCSC in the elastic medium.

CNTs are often embedded in a metal matrix or a polymer in many applications; thus, the dynamic behaviors of the embedded CNTs have been widely investigated. Next, considering the effect of the surrounding elastic medium, the phase velocity of the flexural wave with different frequencies is shown in Figure 9. The stiffness coefficients $c_{13}$ of medium *A* and medium *B* are 184 GPa and 94 GPa, respectively. Figure 9 shows that the phase velocity of the SWCNT in the elastic medium is much higher than that of the independent SWCNT, and the difference between them is more obvious at the lower wave frequency. Similarly, the surrounding elastic medium has significant effects for the lower branches of the CCSC. However, for a given CCSC embedded in a different elastic medium, the width of the bandgap is almost constant, and the effect of the surrounding elastic medium is much weaker for the higher branches.

## 5. Conclusions

In summary, the THz flexural wave propagation in MCSCs have been studied using the nonlocal Timoshenko beam model and MD simulations. The major conclusions are summarized as follows:

(1) For flexural wave propagation in MCSCs, two branches of wave dispersion relation are obtained. Taking the CCSC as an example, the MD simulation shows that the wave could hardly propagate in the CNT when the frequency is within the bandgap between the two branches. Moreover, the phase velocity of the CCSC is close to that of the SWCNT at a lower frequency, and the difference between the CCSC and the SWCNT becomes obvious when the frequency is close to the bandgap.
(2) The effect of the surrounding elastic medium for the width of the bandgap can be ignored. However, it has remarkable effects for the phase velocity at a lower wave frequency.
(3) The phase velocity and the width of the bandgap can be adjusted by changing the mass chain and the stiffness coefficients of the surrounding elastic medium.
